# Establishment of a recombinase polymerase amplification (RPA) fluorescence assay for the detection of swine acute diarrhea syndrome coronavirus (SADS-CoV)

**DOI:** 10.1186/s12917-022-03465-4

**Published:** 2022-10-11

**Authors:** Xiao Cong, Yujun Zhu, Xinchao Liu, Yuexiao Lian, Bihong Huang, Yinzhu Luo, Youfang Gu, Miaoli Wu, Yue Shi

**Affiliations:** 1 College of Animal Science, Anhui Science and Technology University and Anhui Province Key Laboratory of Animal Nutritional Regulation and Health, Fengyang, China; 2 Guangdong Laboratory Animals Monitoring Institute and Guangdong Provincial Key Laboratory of Laboratory Animals, Guangzhou, China; 3 Beijing Biaochizehui Biotechnology Company Limited Daxing District, Qingfengxilu No.29, Beijing, China

**Keywords:** Swine acute diarrhea syndrome coronavirus (SADS-CoV), Recombinase polymerase amplification (RPA), Specificity, Sensitivity, Stability, Repeatability, Point-of-care testing (POCT)

## Abstract

**Background:**

Swine acute diarrhea syndrome coronavirus (SADS-CoV) causes acute vomiting and diarrhea in piglets, leading to significant financial losses for the pig industry. Recombinase polymerase amplification (RPA) is a rapid nucleic acid amplification technology used under constant temperature conditions. The study established a real-time reverse transcription (RT)-RPA assay for early diagnosis of SADS-CoV.

**Results:**

The detection limit of the real-time RT-RPA was 74 copies/µL of SADS-CoV genomic standard recombinant plasmid in 95% of cases. The assay was performed in less than 30 min and no cross-reactions were observed with eight other common viruses that affect swine, including classical swine fever virus (CSFV), porcine reproductive and respiratory syndrome virus (PRRSV), pseudo rabies virus (PRV), swine influenza virus (SIV), seneca valley virus (SVA), transmissible gastroenteritis virus (TGEV), porcine epidemic diarrhea virus (PEDV) and porcine deltacoronavirus (PDCoV). The coefficient of variation (C.V.) values of the two standards dilutions and three positive clinical sample ranged from 2.95% to 4.71%. A total of 72 clinical fecal samples from swine with diarrheal symptoms were analyzed with the developed RT-RPA and quantitative RT-PCR. There was 98.61% agreement between the RT-RPA and the quantitative real-time PCR results.

**Conclusions:**

These results indicated that the developed RT-RPA assay had good specificity, sensitivity, stability and repeatability. The study successfully established a broadly reactive RT-RPA assay for SADS-CoV detection.

**Supplementary Information:**

The online version contains supplementary material available at 10.1186/s12917-022-03465-4.

## Background

Coronaviruses (CoVs) are a family of enveloped, single-stranded, positive-strand RNA viruses classified within the Nidovirales order [1]. Four genera of coronaviruses, including alpha, beta, gamma and delta, have been confirmed to cause subclinical, respiratory and gastrointestinal disease in humans, other mammals and birds [2, 3]. In February 2017, a new intestinal virus of piglets termed swine acute diarrhea syndrome coronavirus (SADS-CoV), was discovered in southern China, which caused acute vomiting and diarrhea in newborn piglets, resulting in mass mortality. These major economic losses caused by the disease are a huge threat to the local pig industry [4–6].

The clinical symptoms caused by SADS-CoV are similar to those caused by the three existing swine enteric coronaviruses (SeCoVs) [7, 8], transmissible gastroenteritis virus (TGEV) [9], porcine epidemic diarrhea virus (PEDV) [10–13] and porcine deltacoronavirus (PDCoV) [14–21]. The SADS-CoV virus also called swine enteric alphacoronavirus (SeACoV)[4] or porcine enteric alphacoronavirus (PEAV) [6] does not relate to the above three viruses, so it is very important to establish a point-of-care testing (POCT) method that can quickly and accurately identify SADS-CoV.

The SADS-CoV virus belongs to the alphacoronavirus genus of the coronavirus family, with a genome size of about 2.7 kb and a gene structure very similar to that of the bat coronavirus HKU-2 strain. It includes nine ORFs including non-structural polymers ORF1a and ORF1b, structural proteins Spike (S), envelope (E), membrane (M) and nucleocapsid (N), as well as auxiliary proteins NS3a, NS7a and NS7 [4, 5, 22].

At present, the main methods for diagnosing SADS-CoV are reverse transcription PCR (RT-PCR) [23], quantitative real-time PCR [24–26], reverse transcriptional loop-mediated isothermal amplification (RT-LAMP) [27, 28], virus isolation culture, immunofluorescence (IF) and immunohistochemistry (IHC), with the gold standard and most common method for the diagnosis of SADS-CoV being nucleic acid testing technology. But some nucleic acid testing methods are time-consuming and complex and require expensive instruments and professional technicians, so it is very important to establish an economical, simple and efficient detection method.

Recombinase polymerase amplification (RPA) is a method that can efficiently amplify DNA at a constant temperature of 37 to 42 °C [29]. In addition, RPA reaction time is shorter at 20 to 30 min compared to RT-LAMP and is a new technique that can be used for clinical and field detection.

The current study is a preliminary examination of reverse transcriptase recombinase polymerase amplification (RT-RPA) fluorescence analysis for the detection of SADS-CoV. The RT-RPA system was found to be accurate, sensitive, specific, and reproducible for point-of-care diagnosis, which would be advantageous for the early diagnosis of diarrhea on pig farms, especially in remote areas and assist the early adoption of effective measures to control the spread of disease.

## Results

### Screening of the primer–probe set

**Fig. 1:**
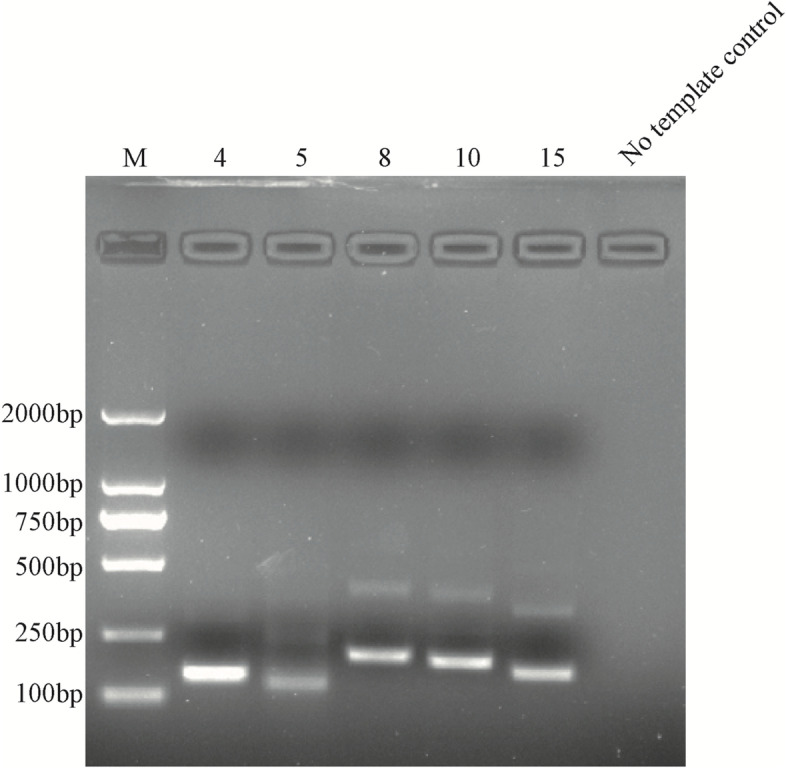
The RT-RPA products, using five primer pairs, were separated via agarose gel electrophoresis. M: Marker; 4: F-4/R-4; 5: F-5/R-5; 6: F-6/R-6; 8: F-8/R-8; 10: F-10/R-10; 15: F-15/R-15

Five pairs of RPA primers were designed for SADS-CoV-M and are listed in Table 1. The extracted viral RNA was used as a template, five primer combinations were amplified using the basic RT-RPA assay and the amplification products were evaluated on 2.0% agarose gels, in Fig. 1.

**Table 1:** Primer and probe sequences used in RT-RPA system

Names	Sequences (5’-3’)	Sizes
F-4	ATTAATAGTTTTAAGCTTTATCGCAGAACG	151 bp
R-4	CACTTAAAATCGTCAGAGTAATACCTGTT	
F-5	AAGTTTTAGAACATCTTAGAAACTGGAACT	130 bp
R-5	CACATGATTAACATCTTAACTCCATAGAGAA	
F-8	TATTACTCTGACGATTTTAAGTGGAACAC	202 bp
R-8	ATAGTCACCATGCTTACTTCTGACATAAAA	
F-10	GATTTTAAGTGGAACACTCTTTTTCGATG	191 bp
R-10	AATAGTCACCATGCTTACTTCTGACATAAA	
F-15	TTAATAGTTTTAAGCTTTATCGCAGAACG	168 bp
R-15	CATCGAAAAAGAGTGTTCCACTTAAAATC	
M-F	ATTGGTGGTTCTTCTCCTTC	316 bp
M-R	ATTATAGTCGTGCCAGGTTT	
Probes	ACGCCATTGCTGTCATTTCAGTCTTTGG[FAM-dT]A[THF]A[BHQ1-dT]CCTACTCGATACC

### Evaluation of RT-RPA system detection performance

#### Specificity and sensitivity

**Fig. 2:**
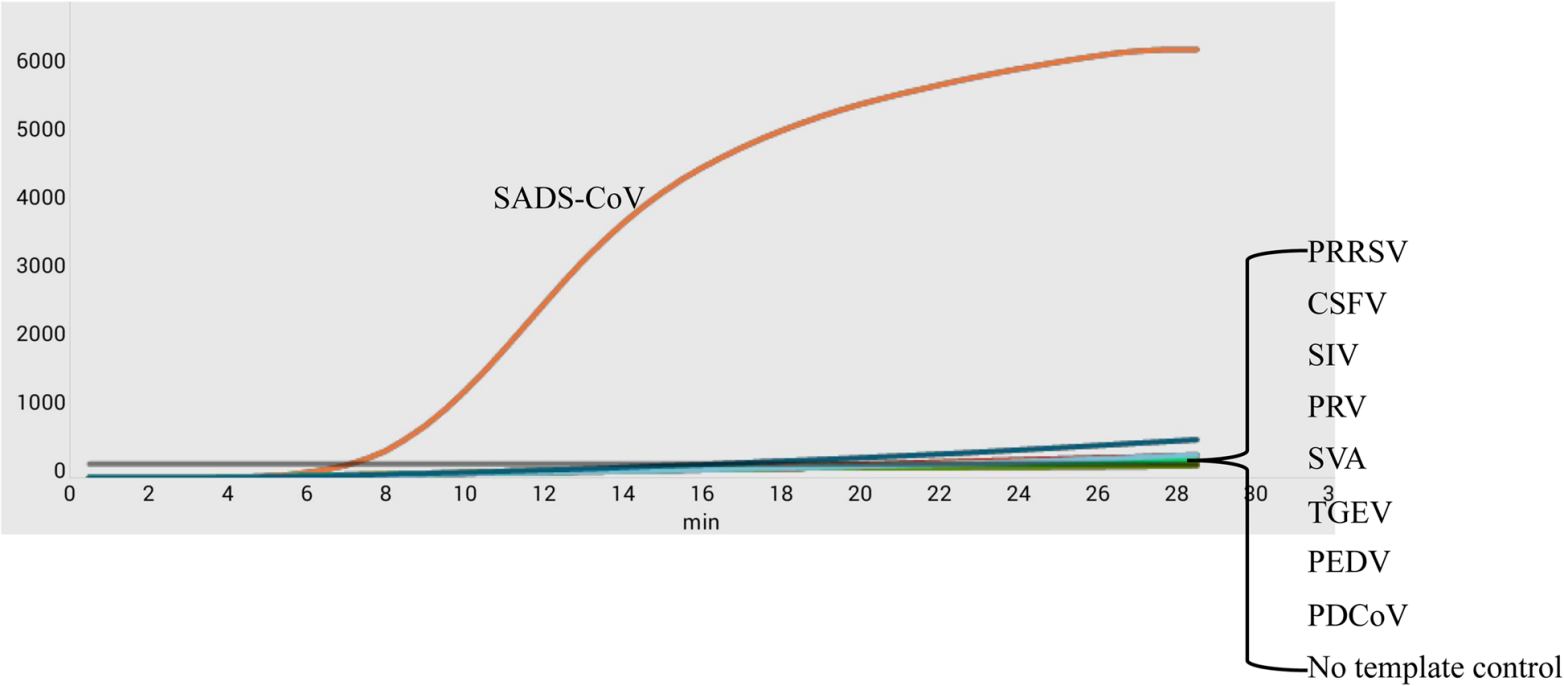
Specificity of the RT-RPA assay was observed. Nucleic acids of CSFV, PRRSV, PRV, SIV, SVA, TGEV, PEDV, PDCoV, SADS-CoV, and control water were evaluated to assess specificity

Nine relevant swine viral nucleic acids, from classical swine fever virus (CSFV), porcine reproductive and respiratory syndrome virus (PRRSV), pseudo rabies virus (PRV), swine influenza virus (SIV), seneca valley virus (SVA), transmissible gastroenteritis virus (TGEV), porcine epidemic diarrhea virus (PEDV), porcine deltacoronavirus (PDCoV) and SADS-CoV were used in this study. As shown in Fig. 2, significant amplification was only observed for SADS-CoV but not the eight other viral nucleic acids and the control water.

**Fig. 3:**
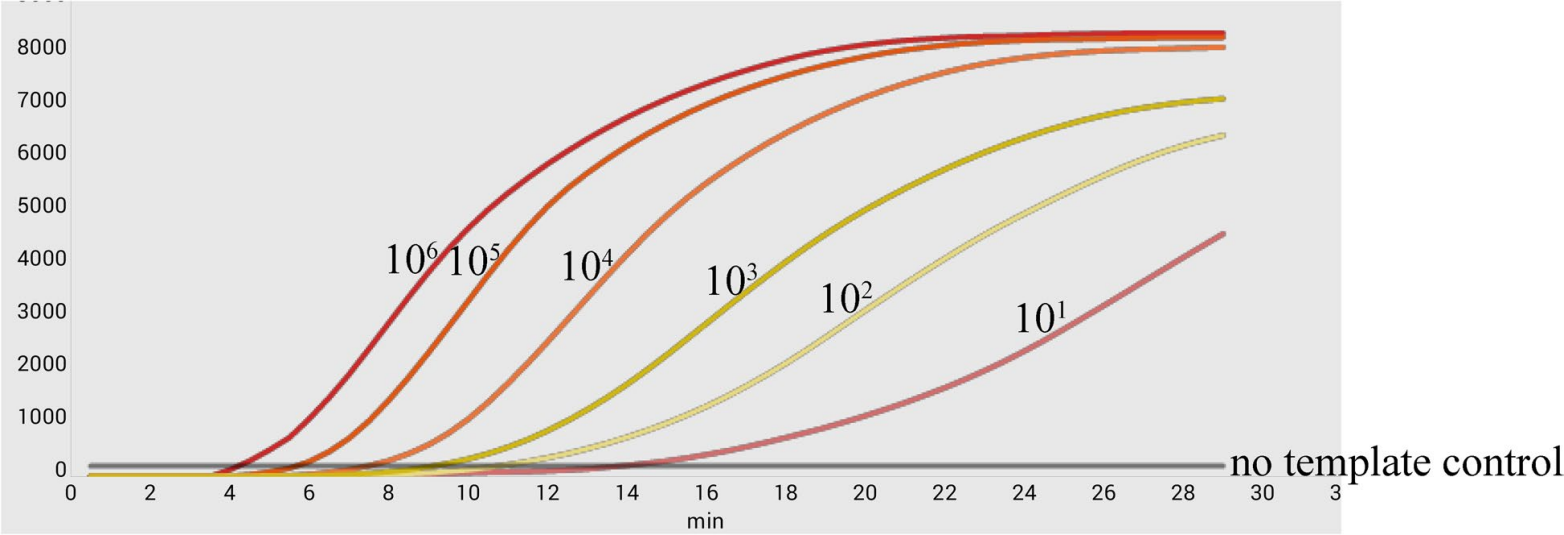
Sensitivity assessment of the RT-RPA assay was performed with 10^6^ to 10^1^ copies/μL standard dilutions

**Fig. 4:**
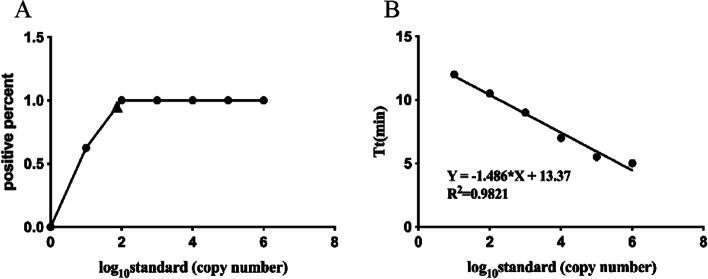
**A** Probit regression analysis using the data from the eight runs. The detection limit at 95% probability (95 molecules) is depicted by a triangle. **B** Semi-logarithmic regression between the Tt values and standard concentrations by GraphPad Prism 7.0

Serial dilution of the standard recombinant plasmid from 10^6^ to 10^1^ copies/μL was initially conducted to investigate the detection limits of the established RT-RPA assay for SADS-CoV. Figure 3 showed that the RT-RPA assay enabled the detection of SADS-CoV within 30 min and the detection limit was maintained at 10^1^ copies/μL. A probit regression analysis using the results of eight runs was performed to determine the exact detection limit and the results showed that RT-RPA could detect 74 copies/μL standard at 95% probability, as seen in Fig. 4-A. The semi-logarithmic regression analysis was performed using the data from the sensitivity test results of standard samples. There was a significant linear relationship between the logarithm of the standard concentration and the Tt value for SADS-CoV where *R*^2^ = 0.9821, as in Fig. 4-B.

#### Stability and reproducibility

To validate the stability of the established RT-RPA detection system, detectable concentrations of standard were diluted to 10^5^ and 10^3^ copies/μL and the two standard dilutions were repeated five times. The C.V. values of the detectable concentration standard samples was less than 5%, as shown in Table 2. The three clinical SADS-CoV-infected fecal samples were each tested five times and the C.V. values were 4.62%, 4.71% and 4.62%, as listed in Table 2, which indicated that the RPA assay was repeatable and precise.

**Table 2:** The mean, SD, and C.V. of Tt values for each of five replicate experiments

Simple	Tt					Mean	SD	C.V. (%)
10^5^ copies/μL	5:30	5:00	5:00	5:00	5:00	5:06	0.2	3.92
10^3^ copies/μL	8:30	8:00	8:00	8:30	8:30	8:18	0.245	2.95
Clinical sample 1	5:30	5:30	5:00	5:30	5:00	5:18	0.245	4.62
Clinical sample 2	5:00	5:30	5:00	5:30	5:00	5:24	0.245	4.71
Clinical sample 3	5:30	5:30	5:00	5:00	5:30	5:18	0.245	4.62

*C.V* SD/Mean*100%

#### Performance of the RT-RPA system in clinical samples

A total of 72 samples made up of 24 intestinal and 48 fecal, were collected by the Guangdong Laboratory Animals Monitoring Institute to evaluate SADS-CoV using the RPA and quantitative real-time PCR assays. The results from testing 10 SADS-CoV positive and 62 negative samples with the RT-RPA system compared with quantitative real-time PCR assays, showed that no negative samples from the quantitative real-time PCR assay were found to be positive by the new RT-RPA system. One sample detected as negative by RT-RPA was detected as positive by quantitative real-time PCR and the Ct value of the sample was 21.174.

The coincidence rate defined as the proportion of the number of samples with the same test results in the total number of test samples between the quantitative real-time PCR and the RT-RPA system was 98.61% for SADS-CoV as shown in Table 3. The sensitivity of RT-RPA for the virus was 90.91% and the specificity was 100%, demonstrating that the RT-RPA system was effective and dependable.

**Table 3:** Comparisons between the RT-RPA system and the quantitative real-time PCR assay for detection of SADS-CoV

		Quantitative real-time PCR			Performance of the RT-RPA system		CR%
		+	−	Total	Sensitivity%	Specificity%	
RT-RPA	+	10	0	10	90.91	100	98.61
	-	1	61	62			
	Total	11	61	72			

*CR* Coincidence rate, *CR* (True positive + True negative)/Total*100%, + Positive, − Negative

**Fig. 5:**
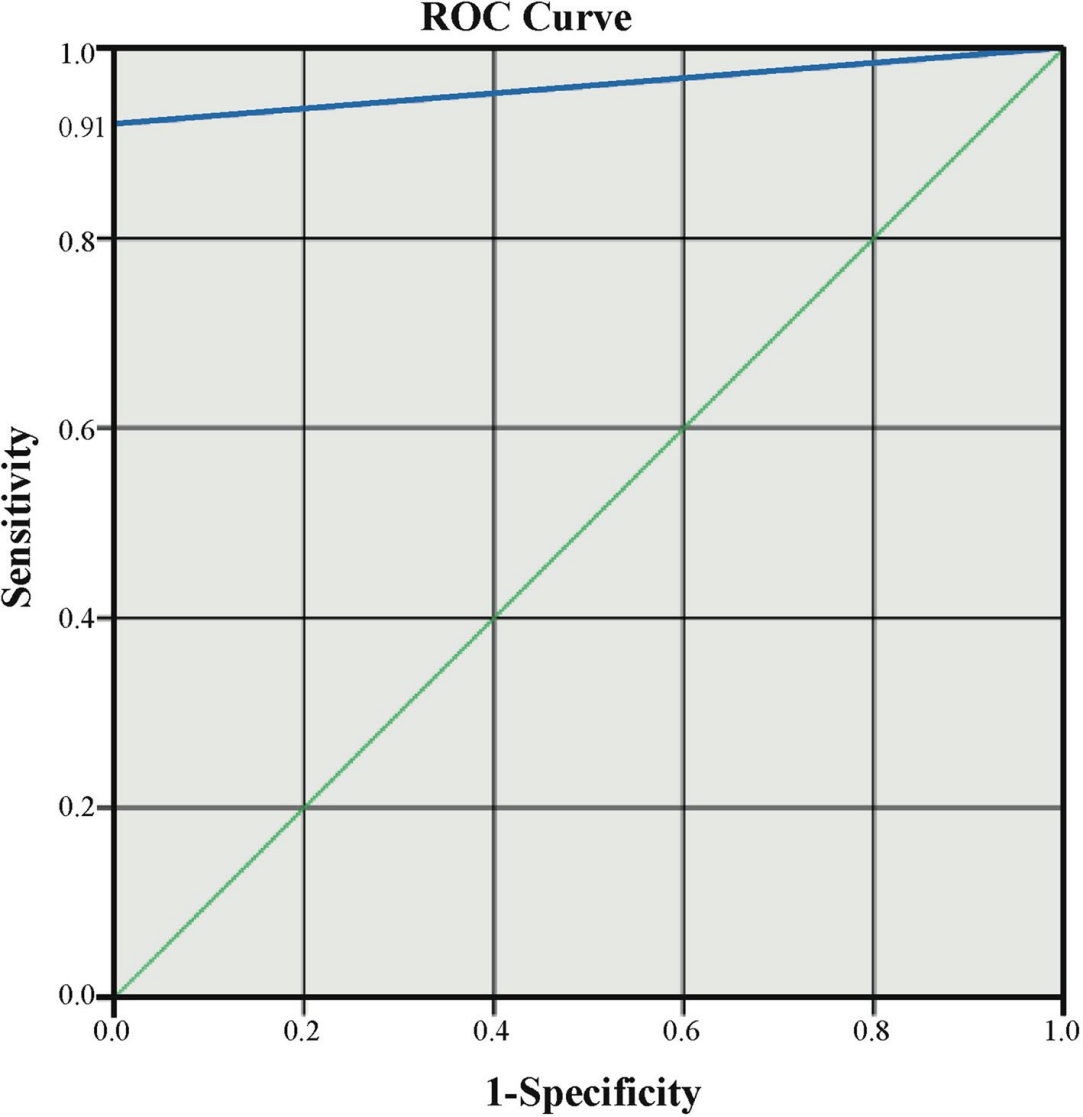
Receiver operating curve (ROC) analysis of the RT-RPA detection system for the diagnosis of SADS-CoV and comparison of the diagnostic accuracy between the RT-RPA detection system and quantitative real-time PCR assay

Based on the above data, ROCs were plotted to determine the diagnostic accuracy of the RT-RPA system and the quantitative real-time PCR assay. The Youden index of the drawn ROCs curve was 0.91 and the corresponding AUC_ROC_ was 0.955. Further analysis showed that there was no significant difference (*p* < 0.0001) between the two methods of the established RT-RPA system for detecting SADS-CoV and quantitative real-time PCR assay, as seen in Fig. 5.

## Discussion

The SADS-CoV virus which is infectious disease, was first identified on Chinese pig farms and therefore may become a significant threat to human and animals’ health. Rapid, convenient diagnosis of SADS-CoV would play a key role in intervention and the implementation of preventive measures. In the present study, a real-time RT-RPA assay was established for rapid and specific detection of SADS-CoV. In a previous report [28], the M protein of SADS-CoV was found to be a highly conserved transmembrane protein and this study designed a primer–probe set based on those M gene sequences.

The RPA technology is ideal for on-site sample testing. The requirements for detection equipment are low, samples can be detected even at human body temperature and the method has high sensitivity, even within 10 copies/μL in some studies [30]. In recent years, the recombinase polymerase amplification lateral flow dipstick (RPA-LFD) detection method has become well known. This method can detect antigens in clinical samples within 10 min, but its sensitivity is lower than that of RT-PCR and quantitative real-time, so it is not suitable for use when the requirements are higher.

The RPA detection can directly use original samples, such as blood, nasal swab, pleural fluid or culture medium, without nucleic acid purification, which saves time in clinical testing and is suitable for high throughput detection [30–32]. Due to its convenience and portability, the RPA technique has been used for the rapid detection of various pathogens, such as feline coronavirus (FCoV) [33], avian reovirus [34], human norovirus [35], murine norovirus [36] and pathogenic bacteria [37], but currently there is no current report about the use of the RPA assay for SADS-CoV monitoring.

An RT-RPA fluorescence assay was developed to amplify the M gene of SADS-CoV. It detected SADS-CoV within 15 min at 42 °C, with a detection limit of 74 copies/μL and did not cross-react with CSFV, PRRSV, PRV, SIV, SVA, TGEV, PEDV and PDCoV. There was 98.61% agreement between the RT-RPA and the quantitative real-time PCR results and further analysis showed that there was no significant difference (*p* < 0.0001) between the two methods. It therefore provided the possibility of rapid clinical detection of SADS-CoV infection and had the potential to detect SADS-CoV in directly boiled animal tissue or stool samples in the future. This procedure would be valuable to reduce sample processing time and increase portability.

## Conclusions

This study established a real-time fluorescence-based RT-RPA assay, which can detect SADS-CoV within 15 min and has a detection limit of 74 copies/μL. The SADS-CoV RPA does not cross-react with CSFV, PRRSV, PRV, SIV, SVA, TGEV, PEDV, or PDCoV. Compared with quantitative real-time PCR, this assay is convenient and less labor intensive and it provides the possibility for rapid clinical detection of SADS-CoV infection in a variety of samples.

Although RT-RPA fluorescence analysis technology is simple, rapid and highly specific, it is prone to be contaminated during operation, so it is necessary to standardize operations, keep reagents properly and separate the sample addition area from the amplification area.

## Methods

### Clinical samples, virus collection, and nucleic acid extraction

Clinical samples including feces and intestinal contents, were collected from breeding farms in southern China in accordance with the recommendations of National Standards for Laboratory Animals of the People’s Republic of China (GB149258-2010). Samples were preserved at − 80 °C in Guangdong Laboratory Animals Monitoring Institute from the time of receipt until use. Nine kinds of porcine viruses, including classical swine fever virus (CSFV), porcine reproductive and respiratory syndrome virus (PRRSV), pseudo rabies virus (PRV), swine influenza virus (SIV), seneca valley virus (SVA), transmissible gastroenteritis virus (TGEV), porcine epidemic diarrhea virus (PEDV), porcine deltacoronavirus (PDCoV) and swine acute diarrhea syndrome coronavirus (SADS-CoV) are prepared by this laboratory.

Before RNA extraction, the sample is added to the sample diluent (PBS) and mixed, with the supernatant for nucleic acid extraction. The DNA/RNA of virus and clinical samples including feces and intestinal contents were extracted using a Baybio Biotech automatic nucleic acid extraction and purification instrument (Guangzhou Co., China), according to the Baypure universal magnetic bead virus DNA/RNA extraction kit instructions and were stored in a − 70 °C freezer.

### Primer and probe design

The reference genomic sequences of SADS-CoV-M were downloaded from Genbank with the accession number MK651076. There was currently no software available for RPA primer and probe design [36], so based on the highly conserved M gene nucleic acid alignment, a set of primers and a probe were designed using Primer Premier 5 software (Premier, Canada) and NCBI website (USA) according to the TwistDx Assay Design Manual (TwistDx, UK) and synthesized by Sangon Biotech (Shanghai) Co., Ltd. (Shanghai, China), shown in Table 1.

### Cloning of standard recombinant plasmid

A pair of primers M-F and M-R for the SADS-CoV-M gene nucleic acid fragments were designed and the target region was amplified using a one-step quantitative real-time PCR kit (Takara, China). The reaction conditions were reverse transcription (RT) at 37 °C for 30 min, denaturation at 85 °C for five min, 35 cycles at 95 °C for 30 s, 50 °C for 30 s and 72 °C for 45 s and a final extension step at 72 °C for 10 min. The amplicon was recovered using a DNA gel extraction kit (Beyotime Biotechnology, China). The purified amplicon was cloned into the pMD-18 T vector (TAKARA, USA) and designated as pMD-18 T-SADS-CoV-M. The positive plasmid was sequenced by Sangon Biotech (Shanghai, China), quantified by Nano2000 (GE, USA) and converted to copy number.

### Development of RT-RPA assays

The basic RT-RPA reaction was conducted in a 50 μL volume using the Basic RT-RPA kit (Hangzhou ZC Bio-Sci&Tech, China), which consisted of 41.5 μL of A buffer, 2 μL of template, 2 μL of each 10 μM primer and 2.5 μL of B buffer. The microtubes were immediately placed in the heating block and incubated at 42 °C for 30 min. The RPA product was cleaned using a PCR products Clean Up Kit (Beyotime Biotechnology) to remove inhibitors that might affect the agarose electrophoresis and then separated on a 2% agarose gel. The amplicons were sequenced using an ABI 3730XL Sanger-based Genetic Analyzer (Applied Biosystems, Waltham, MA, USDA).

Real-time RT-RPA assay was performed using the Fluorescent RT-RPA kit (Hangzhou ZC Bio-Sci&Tech, China). The reaction conditions for real-time RT-RPA were the same as for the basic RT-RPA, except that 0.6μL of water was replaced by 0.6 μL of probe and was performed at 39 °C in a Deaou-308C Tubes Canner (DEAOU Biotechnology, China). According to the manufacturer, a sample was confirmed positive if the amplification curve was above three-and-a-half standard deviations (3.5 SD) from the background in the course of a valid time range after 17 to 18 min of amplification. A threshold time range of zero to four min 30 s was used [33].

### System evaluation and validation

#### Specificity

To investigate the specificity, nine viruses, including CSFV, PRRSV, PRV, SIV, SVA, TGEV, PEDV, PDCoV, and SADS-CoV were evaluated. RNase Free ddH2O was used as a no template control.

#### Sensitivity

The standard was tenfold serially diluted from 10^6^ to 10^1^ copies/μL with distilled water and used to evaluate the detection limit of the SADS-CoV real-time RT-RPA fluorescence assay. Each dilution was evaluated in eight replicates. A semi-log regression was performed by plotting the threshold time against the log_10_standard copy numbers using Prism 5.0 software (GraphPad, USA). To determine the analytical sensitivity of the RT-RPA technique, a probit regression was performed using Prism 5.0 software (GraphPad, USA).

#### Stability and repeatability analysis

To confirm the stability and repeatability of the developed RT-RPA systems, standard dilutions with high 10^5^ copies/μL and low 10^3^ copies/μL concentrations were used to evaluate the coefficients of variation (C.V.) of the RT-RPA systems. The assay for Time threshold (Tt) was defined as the reaction time required for a particular sample to reach sufficiently positive signals above the baseline during real-time amplification. The two standard dilutions and three positive clinical samples were repeated five times, and Tt of reaching positivity were recorded.

#### RT-RPA and quantitative real-time PCR for the detection of clinical samples

A total of 72 swine clinical fecal samples consisting of 24 intestinal and 48 fecal, from healthy piglets and piglets with diarrhea symptoms (these piglets are less than 15 days of age), were detected using the RT-RPA system and the results were compared with quantitative real-time PCR to check for any nonspecific amplification. The Ct value represents the number of cycles that the fluorescent signal in each reaction tube takes to reach the set threshold. There is a linear relationship between the Ct value and the logarithm of the initial copy number of the template. The higher the initial template concentration, the smaller the Ct value; the lower the initial template concentration, the larger the Ct value. The results were further evaluated by generating receiver operating characteristic curves (ROCs), using MedCalc statistical software, V19.1.4. The SADS nucleic acid was used as the positive control.

## Supplementary Information

**Additional file 1:** **Supplementary Fig. 1. **Stability test of SADS-CoV for five times replicate experiments using (A) 10^5^ copies/μL and (B) 10^3^ copies/μL concentration standards, respectively.

**Additional file 2:** **Supplementary Fig. 2. **(A-C) Repeatability test of SADS-CoV for five times replicate experiments using three clinical virus-infected samples, respectively.

**Additional file 3:** **Supplementary Table 1.** The detection results of 11 samples by quantitative real-time PCR assays and the RT-PCR system.

## Data Availability

All data generated or analyzed during this study are included in this article and are available from the corresponding author on reasonable request.

## Abbreviations

RPA: Recombinase polymerase amplification
SADS-CoV: Swine acute diarrhea syndrome coronavirus
RT-RPA: Reverse transcriptase recombinase polymerase amplification
CSFV: Classical swine fever virus
PRRSV: Porcine reproductive and respiratory syndrome virus
PRV: Pseudo rabies virus
SIV: Swine influenza virus
SVA: Seneca valley virus
TGEV: Transmissible gastroenteritis virus
PEDV: Porcine epidemic diarrheavirus
PDCoV: Porcine deltacoronavirus
C.V.: Coefficients of variation
CoVs: Coronaviruses
SeCoVs: Swine enteric Coronaviruses
SeACoV: Swine enteric alphacoronavirus
PEAV: Porcine enteric alphacoronavirus
POCT: Point-of-care testing
RT-PCR: Reverse-transcription polymerase chain reaction
RT-LAMP: Reverse transcriptional loop-mediated isothermal amplification
IF: Immunofluorescence
IHC: Immunohistochemistry
SD: Standard deviation
ROCs: Receiver operating characteristic curves
AUCs: Area Under Curve
Tt: Time threshold
