# Supplementary material for: Establishment of a recombinase polymerase amplification (RPA) fluorescence assay for the detection of swine acute diarrhea syndrome coronavirus (SADS-CoV)
Source: BMC Vet Res. 2022 Oct 11;18:369. doi: 10.1186/s12917-022-03465-4 (PMC9552127; doi:10.1186/s12917-022-03465-4)
Supplement: Supplementary file 2 — Additional file 2: Supplementary Fig. 2. (A-C) Repeatability test of SADS-CoV for five times replicate experiments using three clinical virus-infected samples, respectively. [file 12917_2022_3465_MOESM2_ESM.pdf]

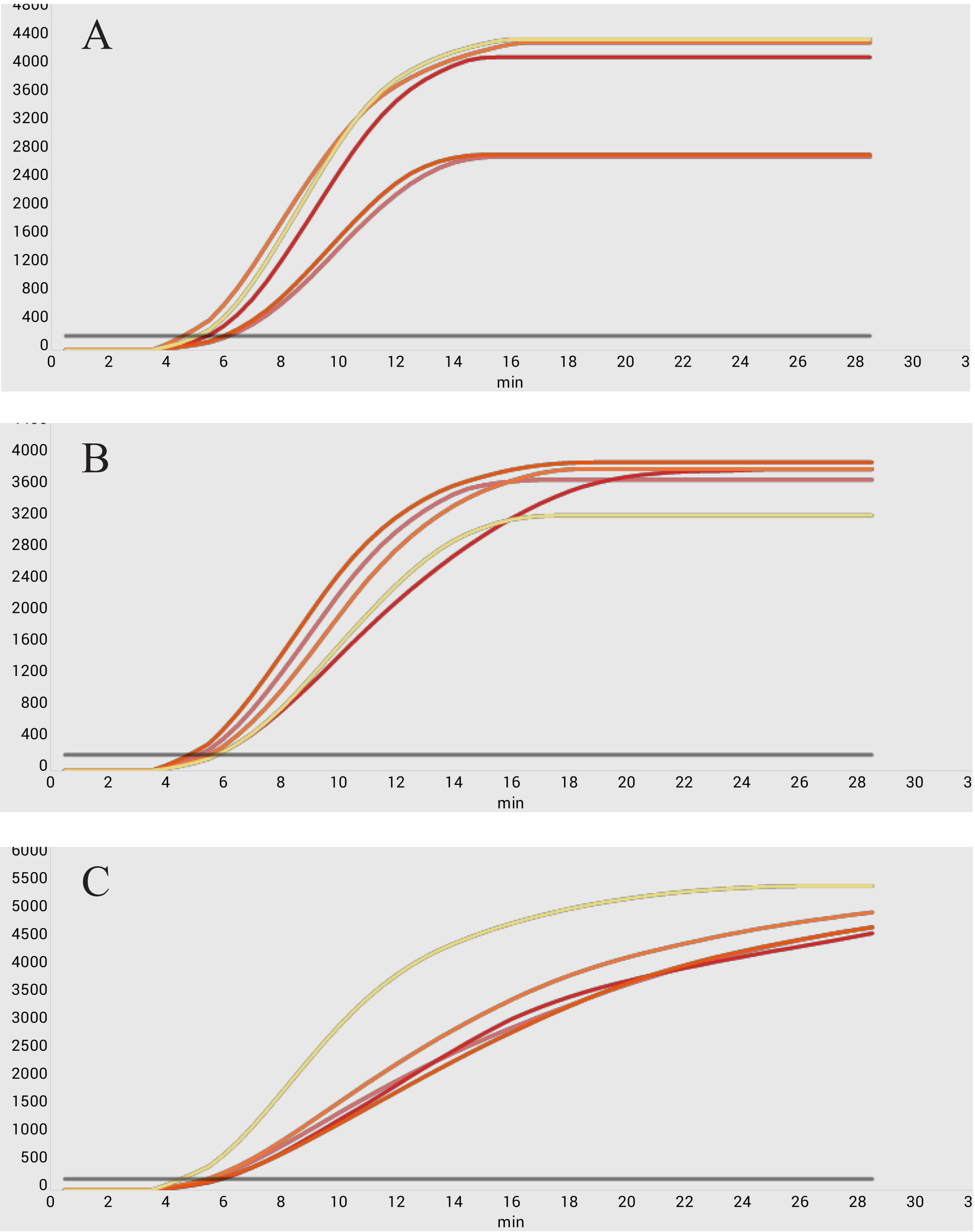

Supplementary Fig. 2. (A-C) Repeatability test of SADS-CoV for five times replicate experiments using three clinical virus-infected samples, respectively.
